# COVID-19 Mortality and Therapeutics in Nebraska and Southwest Iowa during Early Pandemic

**DOI:** 10.3390/pharmacy10040069

**Published:** 2022-06-24

**Authors:** Christopher J. Destache, Faran Ahmad, Sanu Rajendrapasad, Austin Loranger, William Pruett, Nikhal Jagan, Bryan Krajicek, David Schmidt, David Quimby, Manasa Velagapudi, Dayla Boldt, Sarah Hayes, Jennifer Anthone, Brittney Kessel, Renuga Vivekanandan

**Affiliations:** 1 School of Medicine, Creighton University, Omaha, NE 68178, USA; faranahmad@creighton.edu (F.A.); sanurajendrapasad@creighton.edu (S.R.); austinloranger@creighton.edu (A.L.); williampruett@creighton.edu (W.P.); nikhaljagan@creighton.edu (N.J.); bryankrajicek@creighton.edu (B.K.); davidquimby@creighton.edu (D.Q.); manasavelagapudi@creighton.edu (M.V.); renugavivekanandan@creighton.edu (R.V.); 2 School of Pharmacy & Health Professions, Omaha, NE 68178, USA; brittneykessel@creighton.edu; 3 Department of Pharmacy Services, CHI Health, Omaha, NE 68124, USA; david.schmidt503@commonspirit.org (D.S.); dalya.boldt@commonspirit.org (D.B.); sarah.hayes@commonspirit.org (S.H.); jennifer.anthone@commonspirit.org (J.A.)

**Keywords:** COVID-19, therapeutics, remdesivir, steroids, mortality

## Abstract

Different pharmacotherapeutics have been introduced, and then stopped or continued, for the treatment of SARS-CoV-2. We evaluated the risks associated with mortality from SARS-CoV-2 infection. **METHODS:** Data was concurrently or retrospectively captured on COVID-19 hospitalized patients from 6 regional hospitals within the health system. Demographic details, the source of SARS-CoV-2 infection, concomitant disease status, as well as the therapeutic agents used for treating SARS-CoV-2 (e.g., antimicrobials, dexamethasone, convalescent plasma, tocilizumab, and remdesivir) were recorded. Discrete and continuous variables were analyzed using SPSS (ver. 27). Logistic regression identified variables significantly correlated with mortality. **RESULTS:** 471 patients (admitted from 1 March 2020 through 15 July 2020) were reviewed. Mean (±SD) age and body weight (kg) were 62.5 ± 17.7 years and 86.3 ± 27.1 kg, respectively. Patients were Caucasian (50%), Hispanic (34%), African-American (10%), or Asian (5%). Females accounted for 52% of patients. Therapeutic modalities used for COVID-19 illness included remdesivir (16%), dexamethasone (35%), convalescent plasma (17.8%), and tocilizumab (5.8%). The majority of patients returned home (62%) or were transferred to a skilled nursing facility (23%). The overall mortality from SARS-CoV-2 was 14%. Logistic regression identified variables significantly correlated with mortality. Intubation, receipt of dexamethasone, African-American or Asian ethnicity, and being a patient from a nursing home were significantly associated with mortality (x^2^ = 86.36 (13) *p* < 0.0005). **CONCLUSIONS:** SARS-CoV-2 infected hospitalized patients had significant mortality risk if they were intubated, received dexamethasone, were of African-American or Asian ethnicity, or occupied a nursing home bed prior to hospital admission.

## 1. Background

As of 10 June 2022, COVID-19 has infected more than 532 million people globally and caused over 6.3 million deaths worldwide [1]. In the United States, there have been over 84 million confirmed cases and over one million deaths [1]. The COVID-19 pandemic has resulted in devastated economies and stressed the health-care system to breaking point [2].

Researchers and clinicians have extensively studied virus dynamics and host responses from the early days of the pandemic to help discover treatment modalities for COVID-19 [3]. Remdesivir is an antiviral drug with activity against SARS-CoV-1, MERS coronaviruses, and Ebola virus [4,5,6,7]. Dexamethasone has also been shown to improve the outcomes of COVID-19-infected patients in intensive care units [8]. The SARS-CoV-2 illness lasts between 3 and 14 days with an incubation period of 1 to 14 days (most commonly around 5 days) [9]. Typically, COVID-19 patients show the highest viral load near presentation, a prime cause of the fast-spreading nature of this epidemic [10]. Hence, the initiation of potent agents, alone or in combination, needs to be started early in the course of the illness.

Racial and ethnic minorities are infected with COVID-19 more frequently than those of the Caucasian race [11]. The impact of the disease on American communities is not equitable and particularly affects the most vulnerable. Thus, it was felt important to capture the racial characteristics associated with COVID-19 in the rural and urban mid-western United States.

Our objective was to study the racial disparities and to evaluate the time course of the disease and the therapeutics that were used for hospitalized COVID-19 positive patients from March to July 2020. Our health system issued guidance documents based on evolving guidelines for the treatment of suspected and confirmed COVID-19 patients. We sought to measure the mortality of patients admitted to hospitals in our health system (CHI Health; approximately 1000 beds) in east-central Nebraska and western Iowa.

## 2. Methods

TheraDoc (Premier, Inc., Charlotte, NC, USA), the pharmacy clinical decision-support software used in all inpatient CHI facilities in Nebraska and southwest Iowa, was used to identify COVID-19 patients admitted to the health system. Details of patients with positive COVID-19 PCR testing were entered into a spreadsheet and data were gathered from the electronic medical record throughout the hospital stay. The demographic data collected included patient age and weight, APACHE II score and creatinine clearance (CrCl) on admission, and the suspected source of SARS-CoV-2 infection. Concomitant diseases (e.g., cardiovascular disease, COPD, asthma, HIV, diabetes) and outpatient use of ACE or ARB inhibitors were collected. Additionally, details of patient admission location (i.e., general floor or ICU), required intubation, prone positioning, ECMO, and vasopressor support during hospitalization were collected. Details of the therapeutics that were used, including remdesivir, dexamethasone, tocilizumab, hydroxychloroquine (HCQ), and azithromycin were also collected. The initial and maximum C-reactive protein, LDH, D-dimer, ferritin, and fibrinogen levels were also recorded. Finally, the length of ICU and hospital stay, as well as disposition upon discharge (i.e., home, SNF, or death), were also recorded.

In-patients aged 19–95 years who tested positive for COVID-19 in the CHI Health system from 1 March 2020 to 12 July 2020 were eligible for electronic medical record review. After patient details were entered into the Excel spreadsheet, the spreadsheet was locked, and the data were loaded electronically to a statistical software package for analysis. Discrete variables were analyzed using a chi-squared test or Fisher’s exact test and continuous variables were analyzed using a Student’s *t*-test using SPSS-PC (ver. 27).

## 3. Results

CHI Health has hospitals in Grand Island (1), Lincoln (1), and Omaha (5), Nebraska as well as Council Bluffs (1), Iowa. These hospitals admitted patients with a diagnosis of COVID-19 based on a positive SARS-CoV-2 polymerase-chain reaction (PCR) test. A total of 471 patients (admitted from 1 March 2020 through July 12, 2020) comprised our COVID-19 sample. Patients were Caucasian (49%), Hispanic (30%), African-American (14%), or Asian (6.5%) (Table 1). Females accounted for 52% of those infected. A total of 32% of patients in the sample were presumed to have been infected in nursing homes, and 16% in food-processing facilities. The mean (±SD) age and body weight (kg) for COVID-19 positive patients were 62.5 ± 17.7 years and 86.3 ± 27.1 kg, respectively. Sixty-eight percent of patients had a BMI > 25 and were defined as obese.

The admission APACHE II score of patients averaged 9.9 ± 6.5. Twenty-nine percent of patients required an ICU bed and intubation; 19% required prone positioning for management of severe COVID-19 illness. Admission chest X-ray, or computed tomography (CT) of the chest findings, demonstrated bilateral airspace disease in 59% of the COVID-19 patients, unilateral airspace disease in 16%, and minimal airspace disease in 24%.

Hydroxychloroquine (HCQ) use was limited (initiated in 83 (17.8%)), whereas azithromycin was used in 234 (50%) of patients as the initial therapy for presumed community-acquired pneumonia with other antimicrobials. Antibacterial therapy was continued for an average of 5.5 ± 5.3 days. The total length of hospitalization averaged 13.5 ± 12.1 days. The ICU length of stay and length of intubation were 4.7 ± 7.8 and 3.6 ± 7.1 days, respectively. A significant majority of nursing home patients (51%) who acquired COVID-19 died during their hospital stay (*p* < 0.0005). Patients that were categorized as obese or acquired COVID-19 through occupational exposure were not significantly associated with mortality. Finally, significantly higher age was associated with mortality (alive 61.1 ± 17.9 vs. died 72.2 ± 13.1 years, *p* < 0.0005). Our results demonstrated that approximately 5.9% of patients who died came from a rural location (outside Omaha, NE, USA).

A comparison of the initial and maximum inflammatory markers is provided in Table 2. The initial and maximum C-reactive protein (CRP) levels were significantly different for COVID-19 survivors compared to fatalities. Additionally, maximum lactate dehydrogenase (LDH) levels were also significantly different between COVID-19 survivors and fatalities. Initial and maximum ferritin, and d-dimer levels, were not different between survivors and fatalities. The lowest absolute lymphocyte count during hospitalization was not different between survivors and fatalities.

The therapeutic modalities used for COVID-19 illness included dexamethasone (35%), HCQ and convalescent plasma (17.8%), remdesivir (16%), and tocilizumab (5.8%), with some patients receiving all these treatments. Significantly (*p* < 0.001) more patients receiving convalescent plasma or dexamethasone were fatalities from COVID-19. The APACHE II score on admission was significantly higher in patients receiving steroids compared to patients not receiving steroids (13.6 ± 7.1 vs. 9.8 ± 6.7, *p* < 0.001). Additionally, initial and maximum CRP, maximum ferritin, and maximum LDH levels were all significantly (*p* < 0.05) elevated in COVID-19 patients receiving steroids. These results indicate that steroids were used in the patients who were the most critical and had the highest risk of mortality. No mortality benefit was found for remdesivir, HCQ, or azithromycin therapy. Patients receiving tocilizumab for cytokine storm showed significant survival benefit (15% compared to 85% mortality, *p* = 0.001). Additionally, a fatality rate of 25% for COVID-19 patients receiving steroids and tocilizumab was observed, compared to a 9% fatality rate for COVID-19 patients who received neither steroids nor tocilizumab.

COVID-19 patients who received remdesivir + steroids had a 30% fatality rate compared to a 7.8% fatality rate in patients who did not receive steroids and remdesivir. Similar results were found for patients who received convalescent plasma + steroids compared to patients who received neither of these drugs (30.5% vs. 8.6%). A total of eight (1.9%) patients received remdesivir, dexamethasone, and tocilizumab compared to 39 (9.4%) patients who received convalescent plasma, dexamethasone, and tocilizumab. No mortality benefit was found for COVID-19 patients who received combinations of therapeutics. Based on the therapeutic agents used, no differences in mortality were found according to race by univariate analysis.

The majority of patients were able to return home (44.5%) or to a skilled nursing facility, with an overall in-patient mortality from SARS-CoV-2 infection in our health system of 14%.

Binomial logistic regression was performed to understand which variables were significantly associated with mortality from the SARS-CoV-2 illness (Table 3). The variables that were statistically associated with mortality included steroid use, being admitted from a skilled nursing facility, acquiring SARS-CoV-2 from a food-processing facility, and requiring intubation (χ^2^ = 86.36 (13) *p* < 0.0005). The model explained 41.3% of the variation in mortality and correctly classified 84.2% of patients. The sensitivity was found to be 40.4% and the specificity was 93.8%. Intubation, receipt of dexamethasone therapy, Asian ethnicity, and being a skilled nursing facility occupant prior to COVID-19 illness, were significantly correlated with mortality. The area under the receiver operating characteristic (ROC) curve was 0.891 (95% CI 0.851; 0.931), indicating an excellent level of discrimination.

## 4. Discussion

Several investigators have discussed racial disparities associated with COVID-19 viral infection [12,13,14,15]. Approximately 50% of the patients with COVID-19 in our study were from racial minorities, yet the Nebraska and western Iowa population is composed of 5% African-Americans, 2.7% Asians, and 10% Hispanics. Thus, racial disparities are prevalent in rural areas of the United States. Additionally, the death rate from SARS-CoV-2 within our healthcare system appears to be lower than the national average during this time at 20% [16]. This is possibly related to multiple factors, including a better nursing-to-patient ratio compared to north-eastern states, where patient overload has resulted in allocation of patient beds in hallways and temporary care facilities. In addition, there was a time lag in the pandemic waves among different states, with mid-western states lagging behind the coastal states which showed a surge in early cases. This difference in the timing of pandemic waves provided additional time for hospital facilities to become better equipped with treatment and prevention strategies.

Occupational exposure of COVID-19 was associated with 40% of the hospitalized patients in our study. Many essential workers are at heightened risk of acquiring COVID-19 due to difficulties in social distancing in the workplace [17]. These essential workers, including food-processing facility workers often work “elbow-to-elbow” on the processing line without an ability to social distance. Additionally, these workers often do not have the ability to miss work due to mild illness. Finally, these disparities are accentuated as essential workers are less likely to be able to work from home [18]. Our results demonstrated elevated risk of acquiring COVID-19 for such essential workers with 16% of patients acquiring their illness at their food-processing workplace. Additionally, 34% of patients in the sample acquired their COVID-19 infection in a nursing home prior to hospitalization. These associations have been consistently reported in the literature [19,20,21,22]. 

Details of biomarkers associated with COVID-19 were collected upon admission and throughout each patient’s hospitalization. Initial and maximum CRP and maximum LDH levels were significantly higher in COVID-19 fatalities compared to those that survived, suggesting that these could be appropriate biomarkers for monitoring COVID-19 patients. Bivona et al. provided an overview of various biomarkers regarding prognosis and treatment response [23]. They summarized the findings of nine retrospective reviews (total 1395 patients) and a meta-analysis with 2984 patients, all examining the prognostic value of CRP [23]. The meta-analysis concluded that CRP levels were considerably increased in patients with severe and fatal disease [24]. Out of the nine retrospective reviews, seven studies concluded that CRP levels were correlated with disease severity and showed good prognostic accuracy for the assessment of disease severity in COVID-19. However, two retrospective reviews concluded that CRP levels were not a good predictor of disease severity; however, both these studies enrolled considerably smaller numbers of patients (43 and 25 patients only) [25,26]. Similarly, a meta-analysis considering data for 10,491 confirmed COVID-19 patients concluded that elevated CRP and LDH levels were independently associated with increased risk of poor outcomes [27]. Several other studies have noted that LDH levels were significantly higher in ICU patients, reflecting the severity of pulmonary tissue damage [28,29]. The findings of our retrospective study are consistent with the majority of published studies and showed CRP and LDH levels were significantly higher in patients with poor outcomes or mortality. Further research is needed to determine if monitoring these biomarkers produces a survival benefit for COVID-19-infected hospitalized patients.

Two major clinical trials were performed during the same study period as our retrospective analysis (the DisCoVeRy and ACTT-1 trials) [30,31]. Our study population was not a part of either of these trials, but provided instead a real-world retrospective analysis of treatment modalities. The ACTT-1 trial concluded that remdesivir was superior to placebo in reducing the time to recovery for severe COVID-19 hospitalized patients requiring supplemental oxygen. However, the study was not sufficiently powered to detect differences in the benefits to patients requiring high-flow nasal oxygen, non-invasive ventilation (NIV), mechanical ventilation (MV) or extra-corporeal membrane oxygenation (ECMO). Conversely, the DisCoVeRy trial concluded that there was no clinical benefit of remdesivir in patients having symptoms for more than seven days or who were admitted to hospital requiring oxygen. However, this trial may have been at risk of bias, as it was an open-label study, and its 440 participants were also participating in the WHO Solidarity trial. Currently, remdesivir is the only antiviral drug approved by the FDA for the treatment of COVID-19 [32]. Our retrospective analysis did not show any mortality benefit of either HCQ or azithromycin; the NIH guidelines panel also recommends against the use of these agents for the treatment of COVID-19 [33]. Our retrospective review demonstrated a higher number of deaths in sub-groups receiving dexamethasone or tocilizumab; however, these patients were sicker to begin with, which qualified them to receive these medications. The APACHE II score on admission was significantly higher in patients receiving corticosteroids compared to patients who did not receive corticosteroids (13.6 ± 7.1 vs. 9.8 ± 6.7, *p* < 0.001), which indicates indirectly that the addition of dexamethasone to their treatment regimen was performed appropriately. Subsequently, multiple randomized controlled trials have concluded that administration of systemic corticosteroids (e.g., dexamethasone) improves clinical outcomes in COVID-19 patients requiring supplemental oxygen and hospitalization [34,35]. We found significant survival benefits of tocilizumab in patients with cytokine storm; this finding has been supported by subsequent randomized controlled trials on tocilizumab, the REMAP-CAP and RECOVERY trials [36,37]. Both these trials considered the mortality benefits of tocilizumab in patients with rapid respiratory decompensation associated with an inflammatory response [36]. Our results for the use of therapeutics associated with COVID-19 were not part of the clinical trials for dexamethasone, remdesivir, or tocilizumab [8,38,39,40,41]. Instead, our results are representative of the real-world treatment of COVID-19. However, our results for the use of convalescent plasma have formed part of a larger study sponsored by the Mayo Clinic [42,43]. 

Our healthcare system displays evolving treatment guidelines on our intranet for all clinicians to review and adopt based on their patients’ condition and the severity of disease. Additionally, many clinician practice meetings were held to discuss and adopt these treatment guidelines prior to publication on the CHI intranet.

The results of the logistic regression analysis for variables associated with mortality demonstrated statistically significant results for Asian descent, dexamethasone therapy, need for intubation, and being a skilled nursing facility patient. Intubation and being a skilled nursing facility patient were two variables in the model which displayed significant odds ratio associated with mortality (11.94 and 12.07, respectively). Of interest is that being of Asian race had the highest odds ratio of 14.25. The model constructed showed a 93.8% specificity for mortality and correctly classified 84.6% of patients likely to have a mortality event. It has also been reported elsewhere that the risk of mortality with COVID-19 is significantly higher in African-American populations [44]. Asian populations experience similar outcomes to the non-Hispanic white population according to one systemic review [45]. Another study, from New York City, demonstrated that members of the Asian population had 60% higher odds of hospitalization compared to members of the non-Hispanic white population [46]. Finally, several studies, including a meta-analysis of more than 18 million patients with COVID-19, have documented that individuals of black and Asian ethnicity are at increased risk of COVID-19 infection compared to white individuals, and that Asians are at higher risk of ICU admission and death [47,48].

Further study with this model is necessary, but preliminary analysis indicates that it is useful for the prediction of patients at the highest risk of mortality.

There are some limitations to our study. This is a retrospective review of patient outcomes from a mid-west US population and was performed early in the pandemic. Our institution followed guidelines from the Infectious Disease Society of America (IDSA) for treatment of critically ill COVID-19 patients, but the effect of other treatment options, such as baricitinib, were not considered in this study as they were not available during the study period. The doses and duration of dexamethasone were variable as they were at the discretion of the treating physician which will may have also affected the treatment outcomes. We conducted our study prior to the availability of vaccination against COVID-19 which can alter future disease severity and potentially other outcomes in these patients. Another limitation is that we considered the clinical data for a brief duration of time (March to July 2020). However, this study was performed prior to the emergence of variants of concern (VOC). Extending the study duration would have introduced different VOCs which might have confounded the results. However, we have compared our results with the results of studies performed during a similar timeline to our retrospective analysis to provide a reasonable comparison.

## 5. Conclusions

This real-world review of our healthcare system of patients infected with SARS-CoV-2 using logistic regression demonstrated significant odds ratios with respect to mortality for racial background, including being of Asian descent, intubation, dexamethasone use, and occupancy of a skilled nursing facility prior to hospital admission.

## Figures and Tables

**Table 1:** Demographics of COVID-19 Patients on Admission to the Hospital.

Variable	Result (*n* = 471)
Nursing home	148 (32%)
Food-processing facility	74 (16%)
Patient age (yrs)	62.5 ± 17.7
Body weight (kgs)	86.3 ± 27.1
APACHE II score	9.9 ± 6.5
Patient required intubation	134 (29%)
Patient required proning	88 (19%)
Patient required vasopressor use	97 (29%)
Admission CrCl (ml/min)	75.2 ± 49.2

**Table 2:** Inflammatory Markers of COVID-19 patients.

Inflammatory Marker	COVID-19 Survivor	COVID-19 Fatality
Initial CRP *	72.3 ± 76.4	106.6 ± 86.1
Max CRP *	128.7 ± 91.7	200.3 ± 83.5
Initial D-dimer	199.9 ± 1128.5	297.7 ± 888.2
Max D-dimer	390.6 ± 1495.2	625.9 ± 2095.5
Initial ferritin	1032.2 ± 2095.5	875.9 ± 1936.4
Max ferritin	1207.8 ± 1954.7	2042.6 ± 3526.5
Initial LDH	374.0 ± 160.2	369.8 ± 160.0
Max LDH *	467.9 ± 292.2	598.8 ± 330.9
Lowest absolute lymphocytes count	1.2 ± 2.1	0.7 ± 0.7

* *p* < 0.01 (Student’s *t*-test). CRP = C-reactive protein; LDH = lactate dehydrogenase.

**Table 3:** Results of Logistic Regression for Factors Associated with Mortality.

Variable	Exp (B)	95% C.I.
HCQ therapy	0.986	0.221; 4.23
Azithromycin therapy	1.17	0.46; 3.02
Remdesivir	0.97	0.12; 1.07
Convalescent plasma	1.51	0.52; 4.37
Steroid use *	4.70	1.65; 13.46
Requiring intubation *	11.94	4.23; 33.66
Concomitant cardiovascular disease	2.02	0.77; 5.36
Concomitant diabetes	1.80	0.72; 4.48
Obesity	1.69	0.62; 4.62
Food-processing facility	0.54	0.03; 0.90
African-American race *	2.96	0.80; 11.01
Asian race *	14.25	2.17; 93.50
Hispanic race	2.96	0.75; 11.02
Skilled nursing facility prior to admission *	12.07	3.47 41.97
Tocilizumab	3.77	0.96; 14.85
Constant	8.282	

* variables were significant (*p* < 0.05); 95% C.I. = 95% confidence interval. x^2^ = 86.36 (13) *p* < 0.0005.

## Data Availability

The data presented in this study are available on request from the corresponding author.
